# The Treasury Cattle Commission

**Published:** 1881-10

**Authors:** 


The Treasury Cattle Commission.—The Secretary of the Treasury has appointed a commission to investigate the subject of contagious diseases among the cattle of this country. The commission consists of Prof. James Law, of Cornell; Mr. J. H. Sanders, editor of the *National Live Stock Journal*, and Dr. E. F. Thayer, of West Newton, Mass. These are all most competent men, and their selection is creditable to the good judgment of the Secretary.

The commission met first at Saratoga, and there organized, Prof. Law being made chairman, and Mr. Sanders secretary.

The session was also devoted to laying out the work before them. This will consist in preparing quaratine regulations for cattle imported from Europe; in preparing regulations for the disinfection of cars to be used in the export cattle traffic, and in devising means for definitely ascertaining the extent of the infected districts in the United States. The unwarranted discrimination on the part of the Canadian Government against American cattle was also considered, and Dr. Thayer's report upon the contagious cattle disease prevailing in Nova Scotia was read.

A subsequent meeting was held on August 31st. It was here announced, as the result of previous investigations, that decided pleuro-pneumonia existed in Kings, Queens, Westchester, Putnam, and Richmond counties, New York. The disease had also been found in New Jersey, Pennsylvania, Maryland, Virginia and Delaware; but none west of the Alleghanies.

The commission has also issued a circular, addressed to the Governors of States and Territories west of the Alleghanies, warning of the danger of buying calves or stock east and bringing them west.

Further important work may be expected of the able gentlemen composing the commission in question.

